# Papillary and Trabecular Muscles Have Substantial Impact on Quantification of Left Ventricle in Patients with Hypertrophic Obstructive Cardiomyopathy

**DOI:** 10.3390/diagnostics12082029

**Published:** 2022-08-22

**Authors:** Chengzhi Yang, Haobo Xu, Shubin Qiao, Ruofei Jia, Zening Jin, Jiansong Yuan

**Affiliations:** 1 Department of Cardiology and Macrovascular Diseases, Beijing Tiantan Hospital, Capital Medical University, No. 119 South Fourth Ring West Road, Fengtai District, Beijing 100070, China; 2 Department of Cardiology, Fuwai Hospital, National Center for Cardiovascular Diseases, Chinese Academy of Medical Sciences and Peking Union Medical College, Beijing 100037, China

**Keywords:** hypertrophic cardiomyopathy, papillary muscles, trabecular muscles, cardiac magnetic resonance

## Abstract

Patients with obstructive hypertrophic cardiomyopathy (HOCM) have large papillary and trabecular muscles (PTMs), which are myocardial tissue. PTMs are usually excluded from the myocardium and included in the left ventricular (LV) cavity when determining LV mass (LVM) and volumes using cardiac magnetic resonance (CMR). This conventional method may result in large distortion of LVM and other indices. We investigated 74 patients with HOCM undergoing CMR imaging. LV short-axis cine images were obtained. LV contours were drawn using two different methods: (1) the conventional method, where PTMs were included in the LV cavity; and (2) the mask method, which includes the TPMs in the LV myocardium. The LV end-diastolic volume (LV-EDV), LV end-systolic volume (LV-ESV), LV ejection fraction (LVEF), and the LVM were then calculated. Fasting NT-proBNP and CK-MB levels were measured with ELISA. In patients with HOCM, mass of PTMs (MOPTM) was 47.9 ± 18.7 g, which represented 26.9% of total LVM. Inclusion of PTMs with the mask method resulted in significantly greater LVM and LVM index (both *p* < 0.0001) in comparison with those measured with the conventional method. In addition, the mask method produced a significant decrease in LV-EDV and LV-ESV. LVEF was significantly increased with the mask method (64.3 ± 7.9% vs. 77.2 ± 7.1%, *p* < 0.0001). MOPTM was positively correlated with BMI, septal wall thickness, LVM, LV-EDV, and LV-ESV. LVEF was inversely correlated with MOPTM. In addition, MOPTM correlated positively with NT-proBNP (r = 0.265, *p* = 0.039) and CK-MB (r = 0.356, *p* = 0.002). In conclusion, inclusion of PTMs in the myocardium has a substantial impact on quantification of the LVM, LV-EDV, LV-ESV, and LVEF in patients with HOCM. The effects of the PTMs in women was greater than that in men. Furthermore, the MOPTM was positively associated with NT-proBNP and CK-MB. The PTMs might be included in the myocardium when measuring the LV volumes and mass of patients with HOCM. At present, the clinical and prognostic meaning and relevance of the PTMs is not clear and should be further studied.

## 1. Introduction

Affecting possibly 1/200 of the general population, hypertrophic cardiomyopathy (HCM) is a primary inherited myocardial disorder characterized by unexplained left ventricular (LV) hypertrophy [1]. The LV outflow tract obstruction (LVOTO) is present in about two-thirds of HCM patients, namely obstructive HCM (HOCM). Two principal mechanisms are responsible for LVOTO: (1) septal hypertrophy with narrowing of the LV outflow tract; and (2) anomalous papillary muscles and mitral valve apparatus that are susceptible to the abnormal outflow tract [1]. The LV mass is increased in about 80% of patients with HOCM, which is a predictor of adverse clinical outcomes [2]. Although echocardiography is usually used to assess HOCM, it may miss some hypertrophic segments and underestimate the extent of hypertrophy in the basal anterolateral wall due to limited echocardiographic windows [3]. Owning to its high resolution delineation of hypertrophy, cardiovascular magnetic resonance imaging (CMRI) has been shown to provide precise and reproducible assessment of the LV mass (LVM) and volumes [4].

The papillary and trabecular muscles (PTMs) are myocardial tissue, and thus should be included in the myocardium when determining the LV volumes and mass [5]. However, the PTMs are usually excluded from the myocardium but included in the LV cavity in daily practice in most hospitals, due to the lack of an evaluation approach, and also to the fact that their inclusion makes the analysis too time consuming [6]. This conventional method for the CMRI analysis may be feasible in patients with coronary artery disease or dilated cardiomyopathy because the PTMs contribute little to overall LVM. However, the PTMs may account for a large portion of the LVM in patients with HOCM [7], and the LV cavity becomes smaller in HOCM [8]. Therefore, inclusion of the PTMs in the myocardium or not could have a substantial impact on quantification of the LVM, volumes, and LV ejection fraction [9]. A prior study showed that elevated LVM index (LVMI) was an independent predictor of adverse clinical endpoints in patients with HCM [10]. Furthermore, an extended septal myectomy including abnormally positioned papillary muscles may be essential to relieve the LVOTO [11]. However, data concerning the impact of the PTMs on quantification of LV parameters in patients with HOCM are very limited. Moreover, little is known about the relationship between the PTMs and cardiac biomarkers. Therefore, the aim of this study was to explore effects of the PTMs on quantification of LV parameters, and the relationship between the PTMs and biomarkers of heart failure and myocardial damage using CMRI in patients with HOCM.

## 2. Methods

### 2.1. Study Population

We enrolled patients with HOCM who were evaluated in Fuwai Hospital (Beijing, China) from October 2015 to September 2016. The diagnosis of HCM was based on a maximum left ventricular (LV) wall thickness ≥ 15 mm (or ≥13 mm with an unequivocal family history of HCM), as measured by echocardiography or cardiac magnetic resonance imaging (CMRI), in the absence of other cardiac or systemic diseases capable of producing comparable magnitude of hypertrophy [12]. Evaluation of patients included complete medical history, physical examination, 12-lead electrocardiography, 24-h ambulatory electrocardiographic monitoring, transthoracic echocardiography, blood examination, CMRI, and coronary angiography. The presence of left ventricular outflow tract (LVOT) obstruction was defined as an instantaneous peak Doppler LVOT gradient ≥ 30 mm Hg at rest or during physiological provocation, such as during the Valsalva maneuver, standing, and exercise. Patients with valvular heart disease, coronary artery disease (epicardial coronary stenosis > 70% on coronary angiography, previous myocardial infarction, bypass surgery, or percutaneous coronary intervention), or permanent mechanical device implantation were excluded. Finally, a total of 74 patients with HOCM were recruited in the present study. The protocol of this study was approved by Fuwai Hospital (Beijing, China) ethics committee on 27 September 2016. Informed consent was obtained from all participants.

### 2.2. CMR Imaging

The CMRI was performed using a 1.5-T speed clinical scanner (Magnetom Avanto; Siemens Medical Solutions, Erlangen, Germany) or a 3.0-T scanner (Ingenia, Philips Healthcare, Eindhoven, The Netherlands). The imaging protocol has been described previously [8,13]. First, three orthogonal planes were acquired to localize the heart within the chest. Retrospective electrocardiographic gating cine images were acquired in 3 long axis views. Then, a true fast imaging with steady-state precession (TrueFisp) sequence was used to obtain cine images, which encompassed the LV 2-chamber and 4-chamber long-axis view, the LVOT view, and LV short-axis views (contiguous slices from base to apex for full coverage of the LV). Typical imaging parameters were as follows: field of view (FOV) 360 × 315 mm^2^, repetition time/echo time = 30.9/1.2 ms, slice thickness 6 mm, inter-slice gap 2 mm, image matrix 192 × 162, and flip angle 70°. Patients were asked to hold their breath while images were being acquired.

### 2.3. CMRI Analysis

All the CMRI analysis was performed using a commercial software (QMass MR version 2.1.12.2-×64; Medis Medical Imaging systems, Leiden, The Netherlands) by an experienced radiologist who was blinded to patients’ clinical data. End-diastole and end-systole were identified on the basis of the respective image frames showing the largest and smallest cavity size.

**Figure 1:**
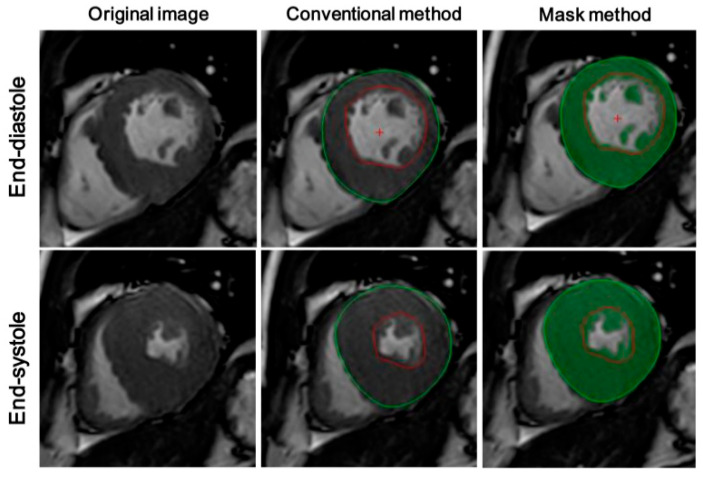
Representative CMR images showing the two methods employed for CMRI analysis. The upper row indicates end-diastolic short-axis view cine images, and the lower row indicates end-systolic images. The middle column demonstrates the conventional method and the third column demonstrates the mask method. In the conventional method, epicardial and endocardial contours of the LV myocardium (excluding papillary and trabecular muscles, PTMs) were traced with the software and corrected manually on each LV short-axis cine image. The area between the green line and the red line indicates myocardium. Then, on the basis of the conventional method, myocardium (including PTMs) was selected with a mask mode using a thresholding algorithm based on the difference of grayscale between the bright blood pool and the dark myocardium. Green parts represent myocardium.

LV contours were drawn using two different methods (Figure 1). (1) The conventional method: Epicardial and endocardial contours of the LV myocardium (excluding papillary and trabecular muscles, PTMs) were traced with the software and corrected manually on each LV short-axis cine image. Hence, PTMs were regarded as part of the ventricular cavity volume. (2) The mask method: On the basis of the conventional method, the myocardium (including PTMs) was selected with a mask mode using a thresholding algorithm based on the difference of grayscale between the bright blood pool and the dark myocardium. The myocardium was selected as the area within the epicardial contours with a pixel signal intensity > 2 SDs of the blood pool (a function of QMass MR version 2.1.12.2-×64; Medis Medical Imaging systems). Manual correction was performed when necessary. Therefore, in this condition, the PTMs were regarded as part of the LV myocardium.

The LV end-diastolic volume (LV-EDV), LV end-systolic volume (LV-ESV), LVEF, stroke volume, cardiac output (CO), and LV mass (LVM) were then calculated in a standard fashion [14]. The LVM was derived by multiplying LV myocardial volume measured at end-diastole with the specific gravity of myocardium (1.05 g/mL). Furthermore, all those parameters were indexed to body surface area, except LVEF. Inter-observer and intra-observer variabilities were tested in a subgroup of 20 randomly selected subjects, for whom two radiologists analyzed CMR images separately.

### 2.4. Laboratory Measurements

Venous blood samples of fasting patients with HOCM were collected within 2 days of echocardiography and 1 week of CMR examination. Plasma levels of N-terminal pro-B-type natriuretic peptide (NT-proBNP) were measured with an electrochemiluminescent immunoassay (Elecsys proBNP II assay, Roche Diagnostics GmbH, Mannheim, Germany) on a Cobas 6000 analyzer (Roche Diagnostics), with a lower detection limit of 0.6 pmol/L. The inter-assay coefficient of variation was <4.6% and the intra-assay coefficient of variation was <4.2%. Serum CK-MB was determined by an immunoinhibition assay (creatine kinase-MB kit, Biosino, Beijing, China) on an Olympus AU-5400 analyzer (Olympus Diagnostics), with a lower detection limit of 3 IU/L. The inter-assay and intra-assay coefficients of variation were <6%.

### 2.5. Statistical Analysis

Continuous variables are expressed as mean ± SD or median (interquartile range (IQR)), according to their normality. Categorical variables are shown as frequencies (percentages). Comparisons of continuous variables between two groups were assessed with a paired Student’s *t*-test. Pearson’s correlation test was used to examine correlations between two continuous variables. Logarithmic transformations were performed for NT-proBNP to obtain normal distribution. Bland–Altman analysis was used for intra-observer and inter-observer reproducibility. A 2-tailed *p* value < 0.05 was considered as statistically significant. Statistical analysis was performed with SPSS version 19.0 software (SPSS Inc., Chicago, IL, USA).

## 3. Results

The clinical characteristics of the 74 patients with HOCM are presented in Table 1. The mean age of patients was 47.0 ± 14.3 years (range 8 to 68), and 47 (63.5%) of the patients were male. All patients had LVOT obstruction. β-blockers were taken by 52 (70.3%) patients, and calcium channel blockers by 24 (25.5%).

**Figure 2:**
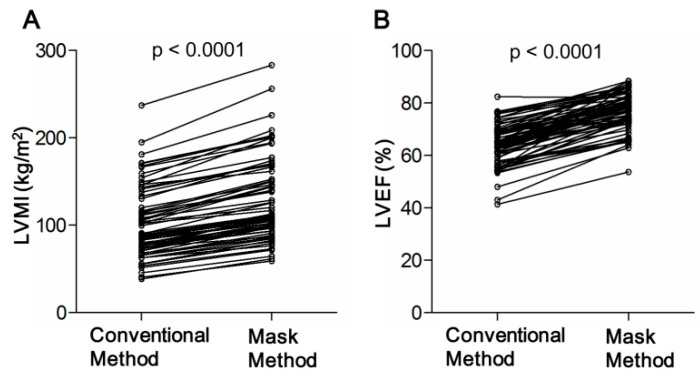
Measurements with the mask method resulted in greater LVMI (**A**) and LVEF (**B**).

The CMRI findings according to the two methods of analysis in patients with HOCM are reported in Table 2. The mass of PTMs, calculated as the LVM measured with the mask method minus that measured with the conventional method, was 47.9 ± 18.7 g, which accounted for 26.9% of the total LVM. Inclusion of the PTMs with the mask method resulted in significantly greater LVM and LVMI (both *p* < 0.0001; Figure 2A) in comparison with those measured with the conventional method. Compared with the conventional method, the mask method produced a 45.5% decrease in LV-EDV, a 128.9% decrease in LV-ESV, and a 20.8% decrease in CO (all *p* < 0.0001). The LVEF was significantly increased with the mask method (64.3 ± 7.9% vs. 77.2 ± 7.1%, *p* < 0.0001; Figure 2B). These findings were present in both male and female patients.

**Figure 3:**
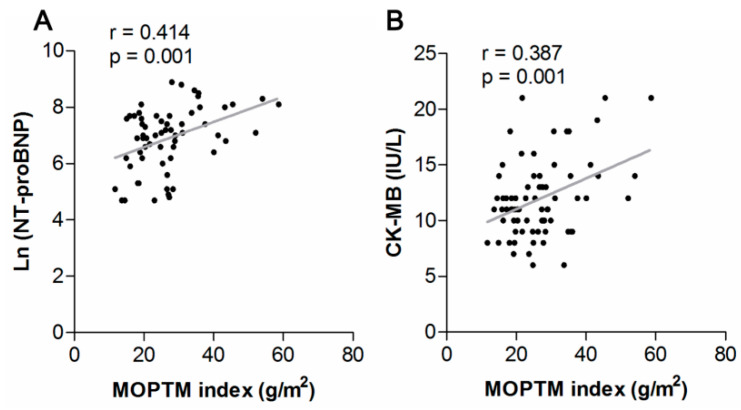
Correlations between mass of papillary and trabecular muscles (MOPTM) index and Ln (NT-proBNP) (**A**), and CK-MB (**B**).

Table 3 shows the correlations between clinical characteristics and the mass of papillary and trabecular muscles (MOPTM) of patients with HOCM. The MOPTM was positively correlated with BMI (r = 0.278, *p* = 0.017), septal wall thickness (r = 0.539, *p* < 0.001), LVM (r = 0.645, *p* < 0.001), LV-EDV (r = 0.719, *p* < 0.001), and LV-ESV (r = 0.767, *p* < 0.001). The LVEF was inversely correlated with MOPTM (r = −0.287, *p* = 0.013). In addition, the MOPTM correlated with Ln (NT-proBNP) (r = 0.265, *p* = 0.039) and CK-MB (r = 0.356, *p* = 0.002). In addition, the MOPTM index was also positively correlated with NT-proBNP (r = 0.414, *p* = 0.001) and CK-MB (r = 0.387, *p* = 0.001) (Figure 3).

**Figure 4:**
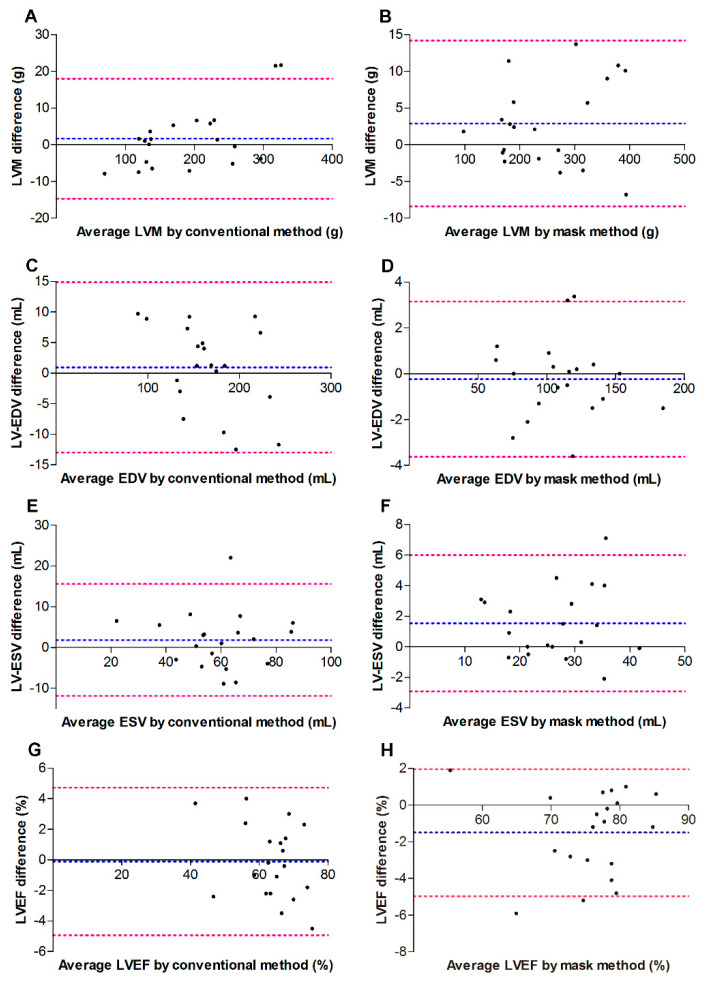
Bland–Altman plots indicating intra-observer agreement for 20 randomly selected subjects. (**A**,**C**,**E**,**G**) indicate data for the standard method. (**B**,**D**,**F**,**H**) indicate data for the standard method. Blue dashed line, bias; red dashed line, 95% limits of agreement between inter-obsever variability. EDV, end diastolic volume; ESV, end systolic volume; LVEF, left ventricular (LV) ejection fraction; LVM, LV mass.

**Figure 5:**
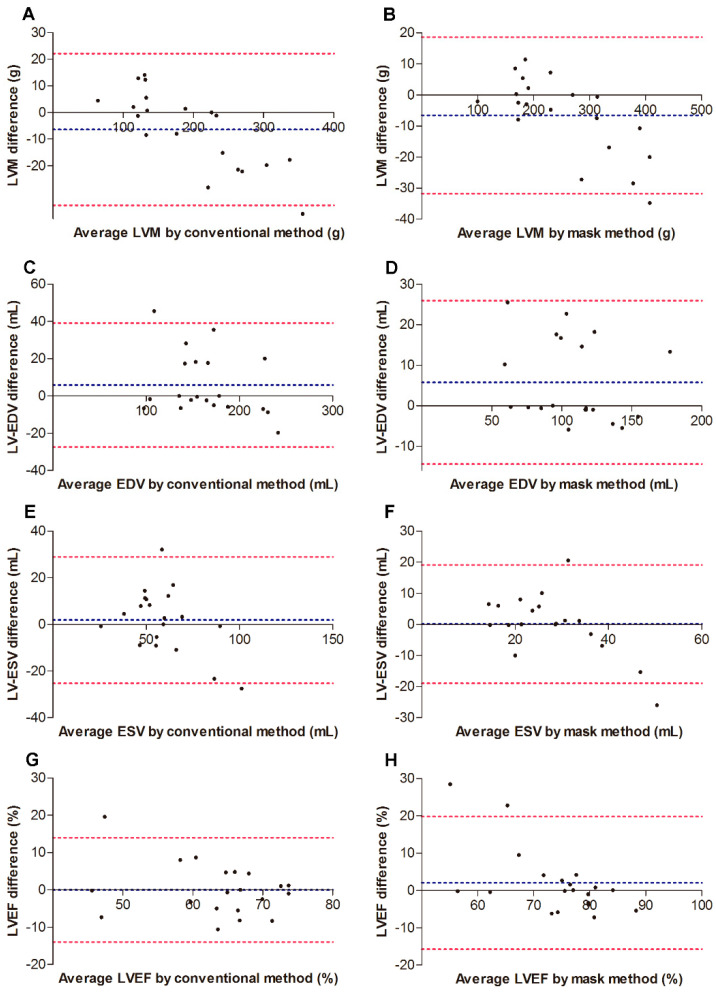
Bland–Altman plots indicating inter-observer agreement for 20 randomly selected subjects. (**A**,**C**,**E**,**G**) indicate data for the standard method. (**B**,**D**,**F**,**H**) indicate data for the standard method. Blue dashed line, bias; red dashed line, 95% limits of agreement between inter-observer variability. EDV, end diastolic volume; ESV, end systolic volume; LVEF, left ventricular (LV) ejection fraction; LVM, LV mass.

The intra-observer and inter-observer reproducibility and variability for the conventional method and the mask method are summarized in Table 4, and in Figure 4 and Figure 5. Regarding intra-observer, the Bland–Altman plots graph showed that the limits of agreement and corresponding 95% confidence intervals (bias) for measurements obtained with the mask method were much narrower than those for measurements obtained with the conventional method. As for the inter-observer, the measurement biases were comparable between the conventional method and the mask method. The Pearson’s correlation test showed that the reproducibility of the LVM and LV-EDV were better than those of LV-ESV and LVEF for both the mask method and the conventional method.

## 4. Discussion

In the present study of 74 patients with HOCM undergoing CMRI, we demonstrated that inclusion of the PTMs in the myocardium using the mask method resulted in greater LVM and LVEF. In addition, the mass of PTMs (MOPTM) correlated positively with the LVM, whereas inversely with the LVEF. Furthermore, the MOPTM index correlated positively with NT-proBNP and CK-MB levels, which reflect severity of heart failure and myocardial damage, respectively.

A few studies have demonstrated that inclusion of the PTMs in the myocardium will lead to greater LVM. A study in a community-based adult cohort performed by Michael L. Chuang et al. showed that the LVM increased by 28% if the PTMs were regarded as myocardium rather than part of the LV cavity [15]. In addition, Gommans et al. investigated the impact of the papillary muscles on the LVM in patients with HCM, and found that inclusion of the papillary muscles resulted in an increase of 8.7% in LVM, which was much less than our data [16]. The LVMI in their study was also less than that of our study. These discrepancies may be due to different managements with respect to the trabecular muscles. The trabecular muscles were not included in the myocardium in their study, whereas both the papillary and trabecular muscles were included in the myocardium in our study. In a study of 30 patients with HCM, Yuchi Han et al. reported that inclusion of the PTMs yielded 17% higher LVMI (117 ± 40 g/m^2^), which is close to our data [17]. The minor discrepancy may result from differences between study populations. All of the patients in our study had HOCM, while Yuchi Han et al. did not provide baseline characteristics of their cohort. They may have some patients with non-obstructive HCM. It was suggested that patients with HOCM had greater mass of the papillary muscles than those who did not have LVOTO [18]. Taken together, our data indicate that the PTMs contribute greatly to overall LVM in patients with HOCM. Given that the LVM is a sensitive risk factor for predicting adverse outcomes in patients with HOCM [2,10], the PTMs should be included in the myocardium when assessing LVM in clinical practice.

Decreased LVEF is an important prognostic marker of mortality in patients with chronic HF [19]. Although most of patients with HOCM have normal LVEF, some patients may have borderline or even decreased LVEF, depending on the stage of disease. Therefore, it is pivotal to measure LVEF accurately in patients with HCOM. In the present study, we found that the conventional method for analysis of CMRI significantly underestimated the LVEF in patients with HOCM compared with the mask method. Inclusion of the PTMs in the myocardium with the mask method produced an increase of 12.9% in LVEF. In a study of patients with LV hypertrophy, the authors showed that inclusion of the PTMs in the myocardium resulted in an increase of 7.1% in LVEF in patients with concentric hypertrophy, whereas the LVEF was increased by 2.4% in patients with eccentric hypertrophy [20]. These data are consistent with ours, in that the HCM is severely concentrically myocardial hypertrophic in geometry. Moreover, in a study of 55 patients with HCM, the authors found that inclusion of the papillary muscles in the myocardium led to a 2.6% increase of LVEF [16], which is less than that in our study. This discrepancy may be partly owing to inclusion of trabecular muscles in the myocardium in our study, while the trabecular muscles were included in the LV cavity in their study. In addition, Eun-Ah Park et al. studied 20 patients with HCM and reported that the LVEF was increased by 16% after the PTMs were included in the myocardium [21], which was higher than our data. Of note, 11 of their patients had apical HCM. Therefore, we speculate that the PTMs have greater impact on LVEF in patients with apical HCM.

In the current study, our data revealed that the LVMI was increased by 30.2% in women and 25.7% in men when the mask method was used. Similar to our findings, Jeanette Schulz-Menger et al. studied 64 HCM patients (33 with HOCM) who were assessed with CMR, and found that the increase in the LVMI from non-obstructive HCM to HOCM was more pronounced in females compared to males [22]. Therefore, the PTMs in women may have a greater effect on the LVM than that in men for patients with HOCM.

Previous studies have revealed that hypertrophic papillary muscles may cause or exacerbate heart failure in patients with HOCM [23]. Our prior study demonstrated that the plasma NT-proBNP and cardiac troponin I (cTnI) levels were elevated in patients with HOCM, owing to heart failure and myocardial injury [24]. In a study of 923 patients with severe aortic stenosis, Elliot J. Stein et al. observed that increments in plasma cardiac troponin T and NT-proBNP were more common as the LV hypertrophy becomes more pronounced, suggesting maladaptive remodeling and cardiac injury [25]. However, the relationship between the MOPTM and biomarkers of heart failure remains unclear. In the current study, we found that the MOPTM index was positively correlated with the NT-proBNP levels, which suggested that the PTMs contributed to heart failure in patients with HOCM. In addition, the MOPTM index was also positively correlated with CK-MB levels, which may indicate injury of hypertrophic PTMs. Our findings may add to evidence for papillary muscle thinning in surgical myectomy for HOCM. Notably, some new molecular biomarkers of cardiac hypertrophy have been reported, such as MiR-96 and growth/differentiation factor 15 [26]. It will be interesting to investigate their roles in HOCM in the future.

It was believed that inclusion of the PTMs in the myocardium was very time-consuming when measuring CMR images [17]. In previous studies where the PTMs were included in the myocardium, contouring of the PTMs usually took a lot of time [21]. However, the mask method employed in our study not only provides accurate quantification of LV mass and volumes, but also does not take too much time on the basis of the conventional method. Notably, over the past 5 years, automatic analysis techniques using artificial intelligence have increased markedly in cardiovascular research. Recently, Qiang Zhang et al. developed a CMR virtual native enhancement (VNE) imaging technology from CMR non-contrast T1 maps and cine images using artificial intelligence [27]. In patients with HCM, the VNE images achieved strong agreement with the late gadolinium enhancement (LGE), increased sensitivity for detecting relatively mild fibrosis regions, and better image quality. Therefore, the VNE technology could replace LGE for faster and cheaper CMR scans [27]. Lately, an algorithm based on deep learning (DL) was employed to automatically quantify right ventricular (RV) ejection fraction (RVEF) from CMR images. The authors reported that the DL algorithm gave rise to substantial and clinically important improvements in patients whose images were difficult to analyze with a previous algorithm [28]. Considering that the geometry of the LV is less complex than that of RV, the artificial intelligence-based deep learning technology may achieve better performance in measuring the LV structure and function in patients with HOCM. The mask method used in the current study is probably more suitable for developing automatic analysis based on deep learning technology than the conventional method.

Regarding the intra-observer and inter-observer reproducibility and variability for the conventional method and the mask method, the Bland–Altman plots in this study indicated that the mask method was comparable to the conventional method; additionally, a Pearson’s correlation test showed that reproducibility of LVM and LV-EDV was better than that of LV-ESV and LVEF for both the mask method and the conventional method. As mentioned above, the LV cavity is smaller in HOCM patients compared with healthy subjects [8]. When the PTMs were excluded from the LV cavity with the mask method in our study, the LV cavity became much smaller than that measured with the conventional method, particularly in the end-systolic phase. Therefore, the variability of LV-ESV is greater than LVM and LV-EDV when the mask method is used. LVEF is calculated as the following formula: (LV-EDV − LV-ESV)/LV-EDV. Therefore, the variability of LVEF is also very large because of the high variability of LV-ESV. Consistently, William E. Moody et al. has reported that the conventional method may offer improved reproducibility but sacrifices accuracy [5].

This study has some limitations that warrant discussion. First, we were not able to address accuracy because the true LV mass and volumes were unavailable. Second, because most of patients enrolled in the present study underwent septal myectomy or alcohol septal ablation after comprehensive assessment, and these invasive therapies may be predominant influence factors of long-term prognosis, we did not assess the influence of the PTMs on prognosis of HOCM. In future investigation, it will be interesting to explore the effects of the PTMs on clinical outcomes in HOCM patients who receive drug therapy, alcohol septal ablation, or septal myectomy.

## 5. Conclusions

Inclusion of PTMs in the myocardium has substantial impact on quantification of the LVM, LV-EDV, LV-ESV, and LVEF in patients with HOCM. The effects of the PTMs in women were greater than that in men. Furthermore, the MOPTM was positively associated with NT-proBNP and CK-MB. The PTMs might be included in the myocardium when measuring the LV volumes and mass of patients with HOCM. At present, the clinical and prognostic meaning and relevance of the PTMs is not clear and should be further studied.

## Figures and Tables

**Table 1:** Baseline characteristics of patients with hypertrophic cardiomyopathy.

Variable	Total (n = 74)	Men (n = 47)	Women (n = 27)
Age, years	47.0 ± 14.3	44.9 ± 12.7	50.8 ± 16.3
NYHA functional class Ⅲ or Ⅳ, n (%)	16 (21.6%)	8 (17.0%)	8 (29.6%)
Body mass index (BMI), kg/m^2^	25.6 ± 3.6	26.2 ± 3.6	24.5 ± 3.3
Systolic blood pressure, mmHg	121.8 ± 15.2	121.7 ± 15.6	121.9 ± 14.9
Diastolic blood pressure, mmHg	73.7 ± 10.4	74.1 ± 10.6	73.0 ± 10.3
Heart rate, beats/min	72.3 ± 11.3	73.4 ± 11.5	67.9 ± 6.7
Hypertension, n (%)	18 (24.3%)	11 (23.4%)	7 (25.9%)
Atrial fibrillation, n (%)	15 (20.3%)	11 (23.4%)	4 (14.9%)
β-Blockers, n (%)	52 (70.3%)	33 (70.2%)	19 (70.4%)
Calcium channel blockers, n (%)	24 (25.5%)	18 (38.3%)	6 (22.2%)
ACEI/ARB, n (%)	6 (8.1%)	4 (8.5%)	2 (7.4%)
LVOTG at rest (mmHg)	81.8 ± 38.2	77.9 ± 30.9	88.3 ± 47.9
NT-proBNP (pmol/L)	1113 (497–2201)	863 (398–1646)	2261 (1032–3592)
Ln (NT-proBNP)	6.9 ± 1.1	6.6 ± 1.0	7.4 ± 1.1
CK-MB (IU/L)	12.0 ± 3.5	12.2 ± 3.4	11.6 ± 3.6

ACEI, angiotensin-converting enzyme inhibitor; ARB, angiotensin receptor blocker; LVOTG, LV outflow tract gradient; NT-proBNP, N-terminal pro-B-type natriuretic peptide. Data are expressed as mean ± SD, number (percentage), or median (interquartile range).

**Table 2:** CMRI findings according to two methods of analysis in patients with hypertrophic obstructive cardiomyopathy.

	Total			Men			Women		
	Conventional Method	Mask Method	Relative Difference	Conventional Method	Mask Method	Relative Difference	Conventional Method	Mask Method	Relative Difference
LVM	179.5 ± 75.7	227.4 ± 89.0 *	+26.9%	195.5 ± 71.0	244.7 ± 84.1 *	+25.2%	151.8 ± 76.9	197.4 ± 90.7 *	+30.0%
LV-EDV	150.0 ± 38.4	103.1 ± 25.9 *	−45.5%	156.3 ± 36.5	109.4 ± 26.8 *	−30.0%	139.2 ± 39.7	92.1 ± 20.5 *	−33.8%
LV-ESV	53.1 ± 18.2	23.2 ± 8.1 *	−56.3%	55.8 ± 18.7	24.9 ± 7.9 *	−55.4%	48.5 ± 16.5	20.1 ± 7.8 *	−58.6%
CO	6.4 ± 1.9	5.3 ± 1.6 *	−20.8%	6.7 ± 1.9	5.6 ± 1.6 *	−16.4%	5.7 ± 1.8	4.7 ± 1.5 *	−17.5%
LVEF	64.3 ± 7.9	77.2 ± 7.1 *	+20.1%	64.2 ± 8.3	76.7 ± 7.1 *	+19.5%	64.4 ± 7.2	78.0 ± 7.2 *	+21.1%
LVMI	99.4 ± 39.8	126.4 ± 46.6 *	+27.2%	103.5 ± 36.4	130.1 ± 43.4 *	+25.7%	92.1 ± 44.8	119.9 ± 51.9 *	+30.2%
LV-EDVI	82.5 ± 17.1	57.5 ± 12.4 *	−30.3%	82.9 ± 17.8	58.3 ± 13.2 *	−29.7%	81.9 ± 16.3	55.9 ± 10.9 *	−31.7%
LV-ESVI	29.5 ± 9.3	12.9 ± 4.3 *	−56.3%	29.6 ± 9.5	13.3 ± 4.1 *	−55.1%	29.4 ± 9.1	12.3 ± 4.5 *	−58.2%
CI	3.5 ± 1.0	3.0 ± 0.8 *	−14.3%	3.6 ± 1.0	3.0 ± 0.8 *	−16.7%	3.5 ± 1.0	2.9 ± 0.9 *	−17.1%

CI, cardiac index; CO, cardiac output; LV, left ventricular; EDV, end diastolic volume; EDVI, EDV index; EF, ejection fraction; ESV, end systolic volume; ESVI, ESV index; LVM, LV mass; LVMI, LVM index. * *p* < 0.0001 between the conventional method and the mask method of the total population and each gender group.

**Table 3:** Correlation between indices measured with the conventional method and the MOPTM of patients with HOCM.

Variable	MOPTM	
	r	*p* Value
Age, y	0.064	0.588
BMI, kg/m^2^	0.278	0.017
SBP, mmHg	0.154	0.192
Septal wall thickness, mm	0.539	<0.001
LVM, g	0.645	<0.001
LV-EDV, mL	0.719	<0.001
LV-ESV, mL	0.767	<0.001
LVEF, %	−0.287	0.013
Left atrium volume, mL	0.164	0.163
LVOTG at rest, mmHg	0.114	0.342
NT-proBNP	0.265	0.039
CK-MB	0.356	0.002

BMI, body mass index; EDV, end diastolic volume; ESV, end systolic volume; HOCM, obstructive hypertrophic cardiomyopathy; LV, left ventricular; LVOTG, LV outflow tract gradient; MOPTM, mass of papillary and trabecular muscles.

**Table 4:** The intra-observer and inter-observer reproducibility and variability of quantitative analysis with the conventional method and the mask method.

	Conventional Method		Mask Method		*p* Value
	Mean ± SD	r	Mean ± SD	r	
**Intra-observer**					
LVM (g)	1.68 ± 8.34	0.996	2.91 ± 5.76	0.998	0.59
LV-EDV (mL)	0.94 ± 7.10	0.988	−0.24 ± 1.73	0.998	0.48
LV-ESV (mL)	1.84 ± 7.0	0.900	1.54 ± 2.27	0.961	0.86
LVEF (%)	−0.11 ± 2.46	0.964	−1.50 ± 2.30	0.944	0.07
**Inter-observer**					
LVM (g)	−6.43 ± 14.55	0.995	−6.57 ± 12.87	0.996	0.97
LV-EDV (mL)	5.84 ± 17.0	0.927	5.77 ± 10.29	0.949	0.99
LV-ESV (mL)	1.89 ± 13.81	0.773	0.11 ± 9.71	0.696	0.64
LVEF (%)	−0.01 ± 7.13	0.713	2.05 ± 9.06	0.673	0.43

Pearson’s correlation coefficient (r) was used to examine consistency of quantitative analysis of the intra-observer and inter-observer. The *p* value was calculated with Student’s *t*-test to evaluate the bias (mean ± SD) between the conventional method and the mask method.

## Data Availability

The data presented in this study are available on request from the corresponding author. The data are not publicly available due to privacy.
